# Plasmacytoid dendritic cells alleviate atopic dermatitis by suppressing Th2 inflammation via the IFN-α/IFNAR1 pathway

**DOI:** 10.3389/fimmu.2026.1762173

**Published:** 2026-05-29

**Authors:** Jun-rong Zhu, Wei Li, Jin-hua Xu, Shang-shang Wang

**Affiliations:** 1 Department of Dermatology, Hubei Provincial Hospital of Traditional Chinese Medicine (Affiliated Hospital of Hubei University of Chinese Medicine, Hubei Provincial Academy of Traditional Chinese Medicine), Wuhan, China; 2 Department of Dermatology, Huashan Hospital, Fudan University, Shanghai, China

**Keywords:** atopic dermatitis, IFNAR1, IFN-α, plasmacytoid dendritic cells, Th2 response

## Abstract

**Background and objective:**

Atopic dermatitis (AD) is a chronic immune-mediated inflammatory skin disease characterized by persistent pruritus and eczematous lesions. Its core pathophysiology involves Th2-skewed immune responses and epidermal barrier dysfunction. Plasmacytoid dendritic cells (pDCs), known for their potent production of type I interferons, have demonstrated immunomodulatory roles in various diseases, including allergic asthma. However, their specific function and underlying mechanisms in AD remain poorly defined. This study aims to investigate the role of pDCs in AD and elucidate their molecular regulatory pathways.

**Methods:**

We employed an integrative approach combining transcriptomic analysis, flow cytometry, an MC903-induced murine model of AD, *in vivo* expansion of pDCs using FLT3L, targeted depletion of pDCs with the 120G8 antibody, and IFNAR1-deficient mice. These complementary strategies were used to systematically evaluate the distribution, activation status, and immunological function of pDCs in AD, as well as the mechanisms involved.

**Results:**

pDCs were significantly elevated in the peripheral blood of AD patients and were associated with increased expression of Th2 cytokines. In the MC903-induced murine model, pDCs showed dynamic accumulation in skin lesions and draining lymph nodes. Expansion of pDCs by FLT3L markedly alleviated AD-like skin inflammation, suppressed Th2 cytokines (IL-4, IL-13) and IgE production, and enhanced the expression of epidermal barrier proteins. In contrast, depletion of pDCs by 120G8 exacerbated the inflammatory phenotype. Importantly, the anti-inflammatory effects of pDCs were abolished in IFNAR1-deficient mice, indicating that pDCs exert their immunosuppressive function in AD primarily through the IFN-α/IFNAR signaling axis.

**Conclusion:**

This study is the first to systematically demonstrate the immunosuppressive role of pDCs in AD. We show that pDCs negatively regulate Th2 inflammation and maintain barrier integrity via the IFN-α/IFNAR1 pathway. These findings provide a theoretical and experimental foundation for targeting pDCs and their downstream signaling as a novel therapeutic strategy in AD.

## 1 Introduction

Atopic dermatitis (AD) is a chronic, relapsing inflammatory skin disease characterized by intense itching and eczematous lesions, significantly impacting patients’ quality of life (1, 2). Its pathogenesis involves epidermal barrier dysfunction, skin microbiome dysbiosis, and immune dysregulation, with Th2-skewed immune responses playing a central role (3, 4). Dendritic cells (DCs), bridging innate and adaptive immunity, are crucial in AD immunopathology (5). While conventional DCs (cDCs), especially cDC2, are known to promote Th2 polarization (6), the role of plasmacytoid DCs (pDCs) remains unclear and controversial (7, 8).

pDCs are specialized in rapid type I interferon (IFN-α) production upon TLR7/9 activation (9). They exhibit functional plasticity, demonstrating protective, anti-inflammatory roles in settings like wound healing and asthma, yet pathogenic roles in diseases like lupus (10–13). In AD, some evidence suggests pDC reduction in lesions may imply a tolerogenic function (14), but their precise role and mechanisms are undefined.

This study aims to systematically investigate the function of pDCs in AD. We explore their spatiotemporal dynamics and mechanism of action to clarify their potential immunoregulatory role in this Th2-dominated disease.

## 2 Materials and methods

### 2.1 Human subjects and clinical samples

This study enrolled 35 adults with moderate-to-severe AD, diagnosed per Hanifin and Rajka criteria, from the Department of Dermatology, Huashan Hospital, Fudan University. Thirty age- and sex-matched healthy volunteers without atopic or chronic inflammatory diseases were recruited as healthy controls (HCs). The study was approved by the hospital’s Ethics Committee (2024–098) and conducted in accordance with the Declaration of Helsinki (Chinese Clinical Trial Registry ID: ChiCTR2000036523). All participants provided written informed consent.

Peripheral blood and/or lesional/non-lesional skin biopsies were collected from all participants. Detailed demographic and clinical data, including disease severity scores (EASI, SCORAD), were recorded at enrollment.

### 2.2 Animals and AD model

Six- to eight-week-old female BALB/c wildtype and IFNAR1-/- mice were housed under specific pathogen-free (SPF) conditions. All animal protocols were approved by the Institutional Animal Care and Use Committee of Huashan Hospital.

An AD-like model was induced by daily topical application of MC903 (calcipotriol, 6 μL/cm²) to the shaved dorsal skin for 14 consecutive days. The MC903-induced model is a well-established murine model of AD that mimics key features of human disease without the systemic immune bias. This makes it ideal for studying local skin inflammation and the role of pDCs in the draining lymph nodes.

Some mice received intraperitoneal injections of recombinant Flt3L or 120G8 monoclonal antibody every other day starting from Day 2. Control mice underwent sham treatment.

### 2.3 Histopathological analysis

Lesional dorsal skin samples were fixed in 4% paraformaldehyde, embedded in paraffin, and sectioned at 4 μm thickness. Sections were stained with hematoxylin and eosin (H&E). Epidermal thickness was measured using ImageJ software, and inflammatory infiltration was scored semi-quantitatively. All assessments were performed by two blinded investigators.

### 2.4 Preparation of single-cell suspensions

Single-cell suspensions from mouse lesional skin and draining lymph nodes were prepared for flow cytometry. Tissues were minced and enzymatically digested with collagenase IV (1.5 mg/mL) and DNase I (0.1 mg/mL) in RPMI 1640 medium at 37 °C for 45 minutes. The resulting cell suspensions were filtered through a 200-mesh strainer, washed, and resuspended in cold PBS. Cell concentration and viability were determined by trypan blue exclusion.

### 2.5 Enzyme-linked immunosorbent assay

Mouse serum levels of IFN-α and total IgE were quantified using commercial ELISA kits according to the manufacturer’s instructions (BioLegend). Serum was obtained by centrifuging clotted blood at 7,500 rpm for 25 minutes at 4 °C. Absorbance was measured at 450 nm, and concentrations were calculated based on standard curves. All samples were assayed in duplicate.

### 2.6 Quantitative real-time PCR

Total RNA was extracted from mouse lesional skin and draining lymph nodes using TRIzol reagent. RNA quality was assessed by NanoDrop. cDNA was synthesized from 500ng – 1μg of RNA, and qRT-PCR was performed using AceQ qPCR SYBR Green Master Mix on a real-time PCR system. All reactions were run in triplicate. Relative gene expression was calculated using the 2^–ΔΔCt method, with GAPDH as the internal reference gene. Fold changes were normalized to the control group.

### 2.7 Flow cytometry

Single-cell suspensions from human PBMCs and mouse tissues were stained with Zombie NIR™ viability dye and blocked with anti-CD16/32 antibody. Cells were then stained with fluorochrome-conjugated antibodies for surface markers. The human antibody panel included CD3,CD4,CD25,CD127,Foxp3,CD45, HLA-DR, CD11c,and CD123. The mouse panel included CD3,CD4, CD25, CD45, B220, NK1.1, CD11b, CD11c, MHC II, CD103, Siglec-H, and PDCA-1.pDCs were defined as CD45^+^ HLA-DR^+^ CD123^+^ CD303^+^ (human) or CD45^+^ B220^+^ PDCA-1^+^ Siglec-H^+^ (mouse). cDCs were identified as CD45^+^ CD11c^+^ MHC-II^+^, with further subsetting into cDC1 (CD103^+^) and cDC2 (CD11b^+^). Th2 cells were gated as CD45^+^ CD3^+^ CD4^+^ IL-13^+^ or IL-5^+^ following stimulation. Tregs were defined as CD45^+^ CD3^+^ CD4^+^ CD25^+^ Foxp3^+^.

For intracellular cytokine staining, mouse cells were stimulated with a cell stimulation cocktail for 6 hours, followed by fixation, permeabilization, and staining for Foxp3, IL-4, IL-5, IL-13, IL-17A, and IFN-γ. Data were acquired on a BD FACSVerse or Cytek Aurora flow cytometer and analyzed using FlowJo software (v10.0.7).

### 2.8 RNA sequencing

Bulk RNA sequencing was performed on 65 PBMC samples (35 AD patients and 30 HCs). PBMCs were isolated by Ficoll-Paque density gradient centrifugation. Total RNA was extracted using the RNeasy Micro Kit, and samples with RNA integrity number (RIN) ≥ 7.0 were used for library preparation. Strand-specific RNA-seq libraries were constructed using the TruSeq RNA Sample Preparation Kit and sequenced on an Illumina HiSeq 2500 platform (2×100 bp paired-end).

Raw reads were quality-controlled (FastQC), trimmed (Trimmomatic), and aligned to the human reference genome GRCh38 (HISAT2). Gene expression levels were quantified (featureCounts) and normalized as TPM or FPKM. The genes presented in the heatmap were hand-picked based on canonical cell-type-specific markers derived from established literature. Immune infiltrationanalysis to estimate the abundance of immune cell types was carried out with XCell (15).

### 2.9 Statistical analysis

Statistical analyses were performed using GraphPad Prism (v7.0). Normality was assessed using the Shapiro-Wilk test. For two-group comparisons, unpaired Student’s t-test (normal distribution) or Mann-Whitney U test (non-normal distribution) was used. Multiple group comparisons were analyzed by one-way ANOVA with Tukey’s *post hoc* test. Data are presented as mean ± SEM. A P-value < 0.05 was considered statistically significant (*P < 0.05, **P < 0.01, ***P < 0.001).

## 3 Results

### 3.1 pDC- and Th2-associated gene signatures are enriched in AD patient blood

We analyzed the transcriptomic profiles and cellular composition of PBMC from AD patients and HCs. 35 moderate-to-severe AD patients and 30 HCs revealed 483 differentially expressed genes. Heatmap showing expression patterns of selected canonical marker genes for cDC1, cDC2, pDC, Th2, and Treg cells in AD patients and healthy controls. Gene selection was based on established cell-type-specific markers from the literature, combined with observed expression trends between AD patients and HCs.

**Figure 1:**
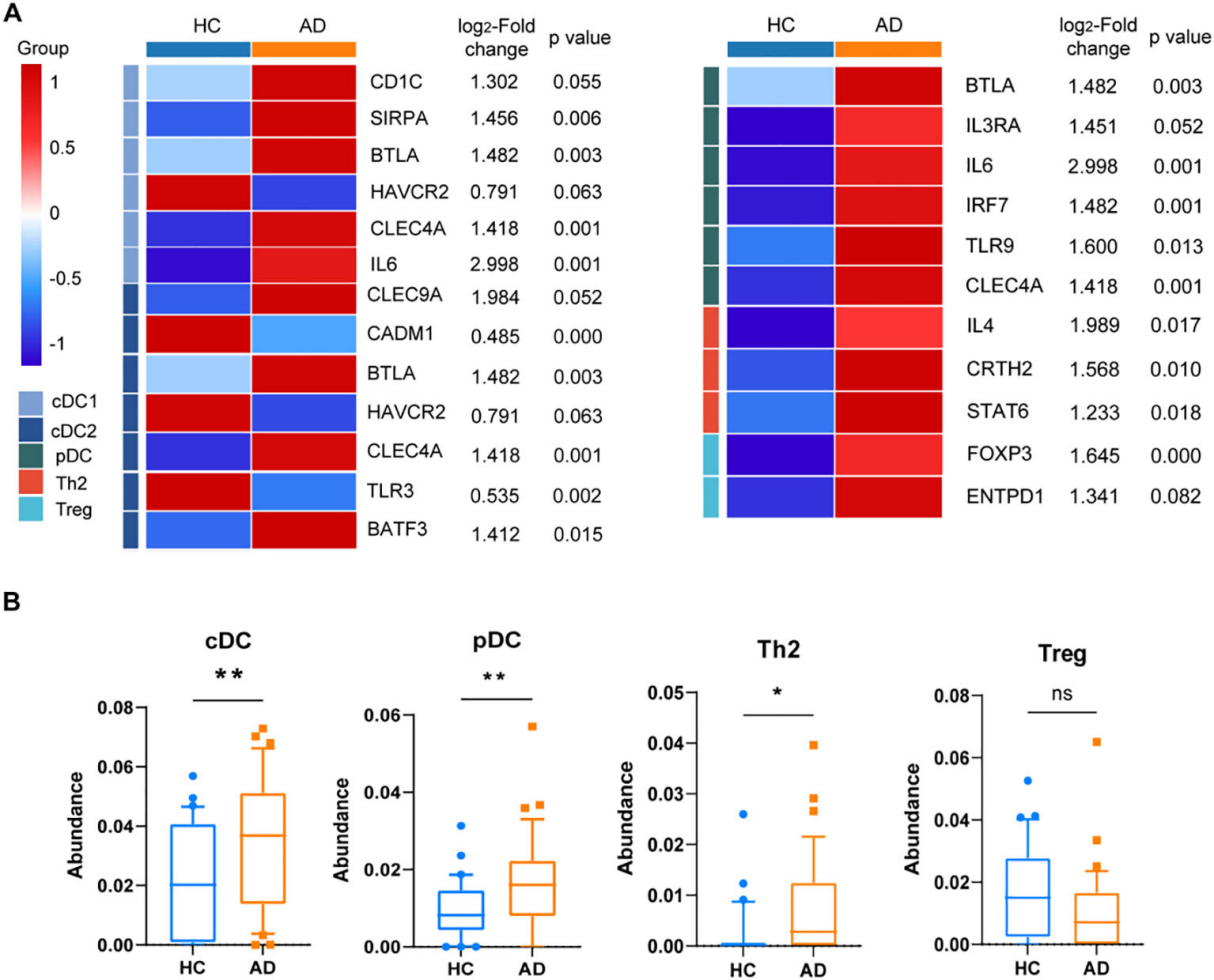
Plasmacytoid dendritic cell (pDC) and Th2-associated signatures are elevated in the peripheral blood of patients with atopic dermatitis (AD). **(A)** RNA sequencing analysis of peripheral blood mononuclear cells (PBMCs) from AD patients (n=35) and healthy controls (HCs, n=30). Heatmap showing the expression patterns of selected canonical marker genes for cDCs, pDCs, Th2 cells, and Tregs. Gene selection was based on established cell-type-specific markers from the literature, combined with observed expression trends between AD patients and HCs.P values were generated by the Friedman test and Dunn’s multiple comparison test. **(B)** Unbiased immune infiltration analysis (xCell) of PBMC transcriptomic data showing estimated abundances of cDCs, pDCs, Th2 cells, and Tregs in AD patients and healthy controls. Boxplots represent the relative abundance scores. The Mann-Whitney U test was used to compare the relative abundance of cells between the two groups. *P < 0.05, **P < 0.01, ***P < 0.001, ns, not significant.

As shown in the heatmap (**Figure 1A**), genes associated with cDC1, cDC2, pDC, and Th2 cells showed an overall trend toward upregulation in AD patients compared to HCs, whereas Treg markers showed variable changes. To more rigorously assess cell-type abundance, we performed unbiased immune infiltration analysis using xCell and updated the results in **Figure 1B**. This analysis revealed significantly higher abundances of cDCs, pDCs, and Th2 cells in AD patients compared to HCs, while Treg abundance was not significantly different between the two groups.

### 3.2 pDCs accumulate dynamically in skin and lymph nodes of AD mice

**Figure 2:**
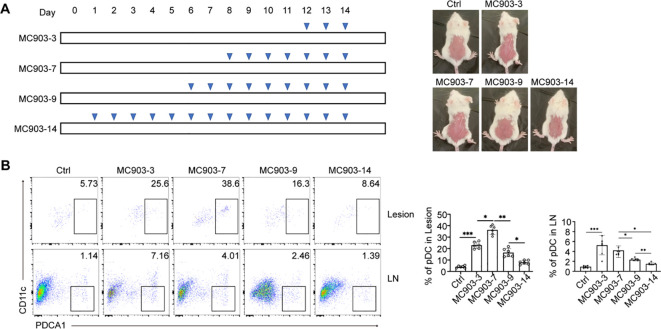
pDCs exhibit dynamic recruitment in the draining lymph nodes and skin of MC903-induced AD model mice. **(A)** Schematic diagram of the experimental timeline, showing daily topical application of MC903 on the dorsal skin of BALB/c mice. Blue triangles indicate the days of topical MC903 application. All mice were sacrificed on the same day. Representative gross appearance of the dorsal skin. **(B)** Flow cytometric analysis of pDC in the lesion and draining lymph nodes (dLNs) over the course of AD development. The proportion of pDCs in the skin lesions peaked on day 7 and gradually decreased thereafter, while in the draining lymph nodes, it reached a maximum on day 3 and then declined. Data are presented as mean ± SEM (n = 5–7 mice per group). *P < 0.05, **P < 0.01, ***P < 0.001 vs. control group.

To investigate the temporal dynamics of pDC during the development of AD, we established an MC903-induced AD mouse model and all mice were sacrificed on the same day(**Figure 2A**). Flow cytometric analysis was performed to assess the proportion of pDCs in the lesion and draining lymph nodes (dLNs) at various time points (gating strategy shown in the material). The results revealed that pDCs significantly increased in the lesion by day 3 after MC903 treatment, peaked on day 7, and gradually declined thereafter; however, their levels remained significantly higher than the control group at the end of the modeling period. In the lymph nodes, pDCs increased markedly by day 3 post-MC903 treatment, and declined thereafter, but remained elevated compared to controls at the study endpoint (**Figure 2B**).

Collectively, these findings demonstrate that pDCs undergo characteristic and dynamic changes in both the dLNs and lesional skin of AD model mice, suggesting their potential involvement in the immunopathogenesis of AD.

### 3.3 FLT3L-induced pDC expansion ameliorates skin inflammation

**Figure 3:**
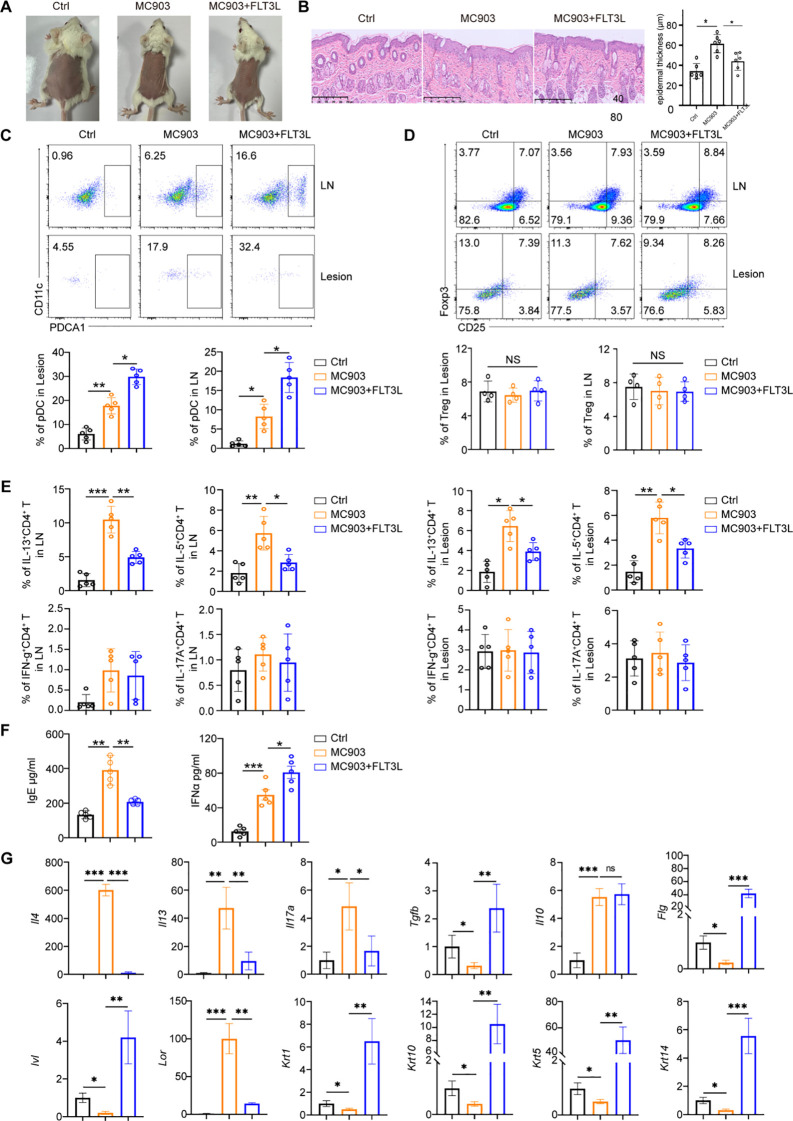
FLT3L-mediated expansion of pDCs alleviates MC903-induced skin inflammation. **(A)** Representative gross images of BALB/c mouse dorsal skin on day 14. **(B)** H&E-stained sections of dorsal skin (scale bar, 100 μm) and quantification of epidermal thickness. Histological analysis confirmed mice in the MC903 group exhibited epidermal hyperplasia whereas FLT3L-treated mice showed reduced epidermal thickness (µm). **(C, D)** Flow cytometric analysis showing the frequency of pDCs and Tregs in lesional skin and dLNs. **(E)** Intracellular cytokine staining showing the frequencies of IL-5^+^, IL-13^+^, IFN-γ^+^ and IL-17A^+^ in skin and dLNs. **(F)** Serum levels of total IgE and IFN-α measured by ELISA. **(G)** qRT-PCR analysis of Th2-associated cytokines (IL-4, IL-13) and skin barrier-related genes (FLG, IVL, KRT1, KRT5, KRT10, KRT14) in lesional skin. Gene expression was normalized to GAPDH and expressed as fold change relative to the control group. Data are presented as mean ± SEM (n = 6–8 mice per group). *P < 0.05, **P < 0.01, ***P < 0.001.

To investigate the functional role of pDCs in AD, we administered Fms-like tyrosine kinase 3 ligand (FLT3L) to MC903-induced mice to promote pDC expansion (16). FLT3L treatment significantly attenuated clinical signs of dermatitis, including redness, scaling, and excoriation (**Figure 3A**), and ameliorated histopathological features such as epidermal hyperplasia (**Figure 3B**). To determine whether the therapeutic effects of FLT3L were specifically mediated by pDCs, we analyzed the composition of other immune subsets in the dLNs. FLT3L treatment resulted in a significant expansion of pDCs and mild cDCs, while the frequencies of macrophages, B cells, and T cells remained unchanged. Representative plots show Treg populations (**Figures 3C, D**).

Functionally, FLT3L treatment suppressed Th2 responses, as evidenced by reduced frequencies of IL-5^+^ and IL-13^+^ cells (**Figure 3E**) and lower serum IgE levels (**Figure 3F**), while enhancing IFN-α production (**Figure 3F**). In contrast, no significant changes were observed in IFN-γ^+^, IL-17A^+^ (**Figure 3E**), suggesting that the protective effect of pDCs is independent of Th1, Th17, or Treg modulation.

qRT-PCR analysis of lesional skin further revealed that FLT3L downregulated Th2-associated cytokines (IL-4, IL-13) and upregulated key barrier-related genes (FLG, IVL, KRT1, KRT5, KRT10, KRT14) (**Figure 3G**).

These data indicate that pDC expansion alleviates AD-like pathology by curbing Th2 inflammation and enhancing barrier integrity.

### 3.4 pDC depletion with 120G8 antibody exacerbates disease

**Figure 4:**
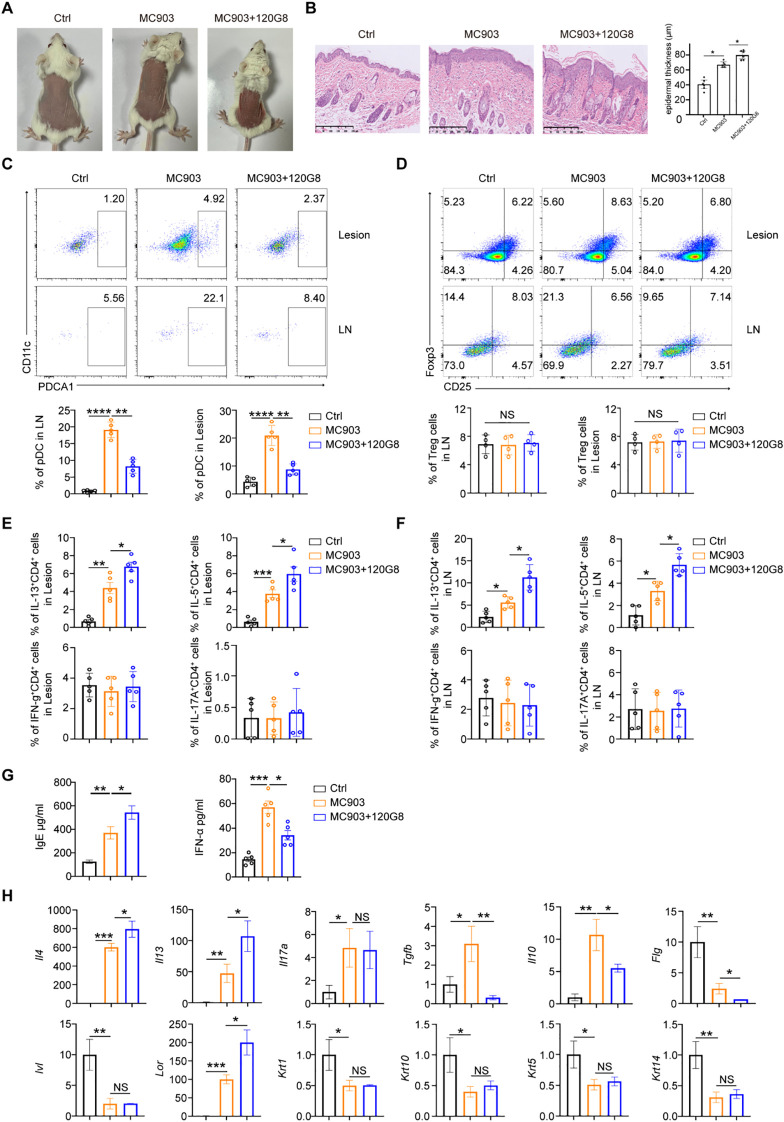
Depletion of pDCs with 120G8 antibody exacerbates MC903-induced AD-like pathology. **(A)** Representative gross images of mouse dorsal skin on day 14. **(B)** H&E-stained sections of dorsal skin (scale bar, 100 μm) and quantification of epidermal thickness. Histological analysis confirmed mice in the MC903 group exhibited epidermal hyperplasia whereas 120G8 antibody exacerbates epidermal thickness (µm). **(C)** Flow cytometric analysis confirming the depletion of pDCs in lesional skin and dLNs following 120G8 administration. **(D)** Representative plots and quantification of Treg populations (CD25^+^ Foxp3^+^) in lesion and dLNs, showing no significant changes following 120G8 administration. **(E, F)** Frequencies of IL-5^+^, IL-13^+^, IFN-γ^+^ and IL-17A^+^ among CD4^+^ T cells in **(E)** lesional skin and **(F)** dLNs, as determined by intracellular cytokine staining. **(G)** Serum total IgE and IFN-α levels measured by ELISA. **(H)** qRT-PCR analysis of Th2-associated cytokines (IL-4, IL-13) and skin barrier-related genes (FLG, IVL, KRT1, KRT5, KRT10, KRT14) in lesional skin. Gene expression was normalized to GAPDH and expressed as fold change relative to the control group. Data are presented as mean ± SEM (n = 6–8 mice per group). *P < 0.05, **P < 0.01, ***P < 0.001.

To further investigate the functional role of pDCs in AD pathogenesis, we administered the pDC-depleting 120G8 monoclonal antibody in MC903-induced AD mice (17). pDC depletion significantly exacerbated MC903-induced skin inflammation, as evidenced by more severe erythema, scaling, accelerated disease progression and histological manifestations (**Figures 4A, B**). Flow cytometry confirmed that 120G8 treatment markedly reduced pDC proportions in both lesional skin and draining lymph nodes (**Figure 4C**) without altering Treg proportions (**Figure 4D**). Functionally, increased serum IgE levels and heightened frequencies of IL-5^+^ and IL-13^+^ cells in skin and lymph nodes indicating 120G8 treatment enhanced Th2 responses. In contrast, no significant changes were observed in IFN-γ^+^, IL-17A^+^ (**Figures 4E–H**).

These findings demonstrate that pDCs play a critical anti-inflammatory role in AD, and their depletion exacerbates disease severity, further supporting a protective function of pDCs in AD pathophysiology.

### 3.5 The protective role of pDCs is mediated by IFN-α signaling

**Figure 5:**
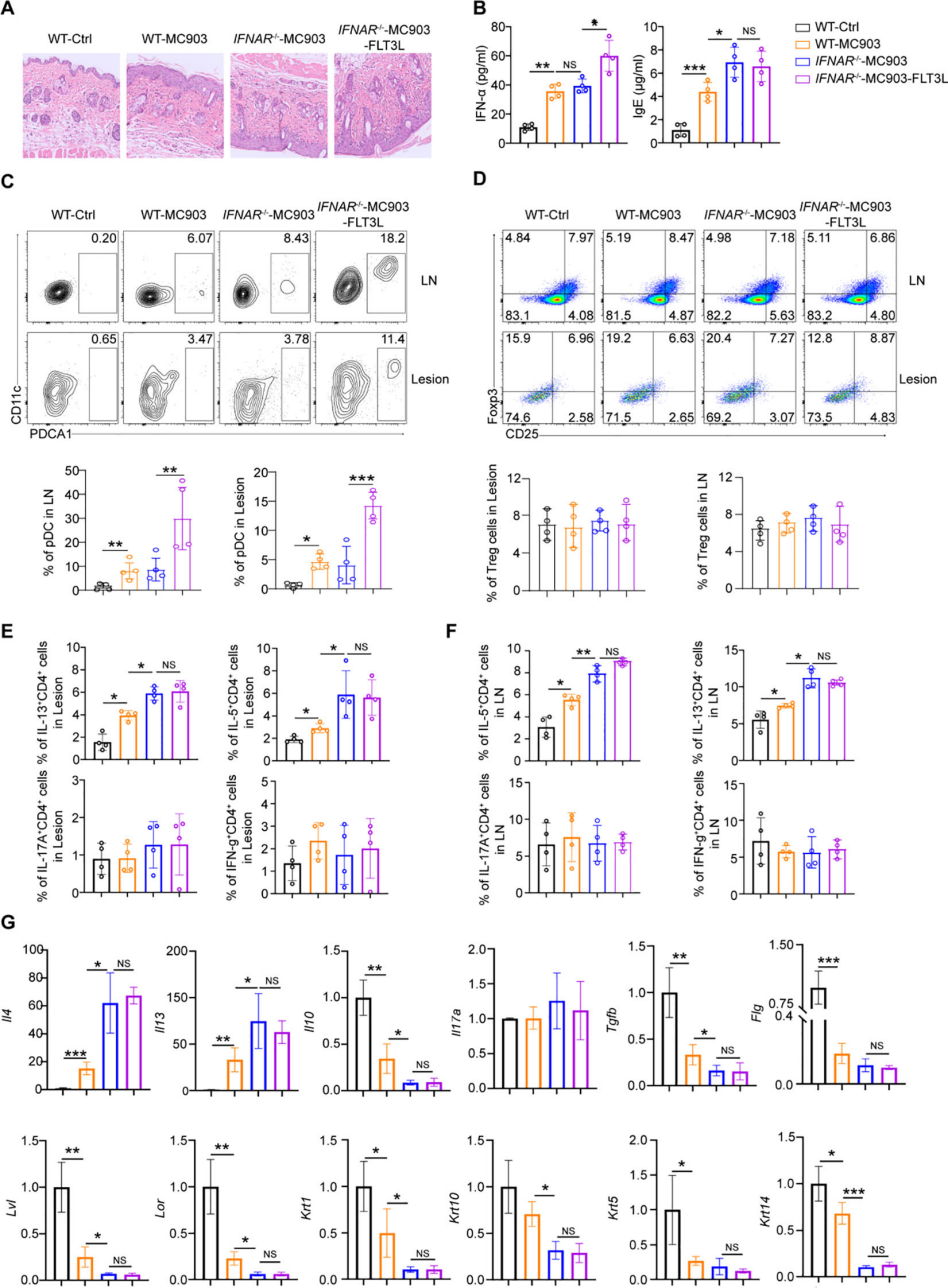
The anti-inflammatory effect of pDCs is dependent on IFN-α/IFNAR1 signaling. **(A)** H&E-stained sections of dorsal skin (scale bar, 100 μm), wild-type (WT) and IFNAR1-deficient (IFNAR1⁻/⁻) mice treated with vehicle (Ctrl), MC903 alone, or MC903 plus FLT3L (MC903+FLT3L) on day 14. **(B)** Serum total IgE and IFN-α levels measured by ELISA in WT and IFNAR1⁻/⁻ mice from indicated treatment groups. **(C, D)** Flow cytometric analysis showing the frequency of pDCs and Tregs in WT and IFNAR1⁻/⁻ mice from indicated treatment groups in lesional skin and dLNs. **(E, F)** Frequencies of IL-5^+^, IL-13^+^, IFN-γ^+^ and IL-17A^+^ among CD4^+^ T cells in (E) lesional skin and (F) dLNs as determined by intracellular cytokine staining. **(G)** qRT-PCR analysis of Th2-associated cytokine genes (IL-4, IL-13) and skin barrier-related genes (FLG, IVL, KRT1, KRT5, KRT10, KRT14) in lesional skin. Expression levels were normalized to GAPDH and shown as fold change relative to the WT control group. Data are presented as mean ± SEM (n = 6–8 mice per group). *P < 0.05, **P < 0.01, ***P < 0.001; n.s., not significant.

To determine whether the protective effects of pDCs in AD require IFN-α signaling, we established MC903-induced AD models in wild-type (WT) and IFNAR1-deficient (IFNAR1⁻/⁻) mice. IFNAR1 deficiency led to more severe skin inflammation, as reflected by elevated serum IgE and increased Th2 cytokines such as IL-5^+^ and IL-13^+^ in lesional skin compared to WT controls (**Figures 5A–F**), indicating a suppressive role for type I IFNs in AD. To assess whether pDCs mediate their effects via this pathway, we expanded pDCs using FLT3L in IFNAR1⁻/⁻ mice. Despite successful pDC expansion, FLT3L treatment failed to attenuate inflammation or reduce Th2 responses in the absence of IFNAR1 (**Figures 5C, E, F**). qRT-PCR analysis confirmed that IFNAR1 deficiency resulted in heightened expression of Th2-associated genes and exacerbated downregulation of skin barrier-related genes following MC903 treatment, with FLT3L unable to restore barrier gene expression in IFNAR1⁻/⁻ mice (**Figure 5G**). Collectively, these findings demonstrate that the anti-inflammatory and barrier-protective functions of pDCs in AD are critically dependent on IFN-α/IFNAR1 signaling.

## 4 Discussion

This study provides a comprehensive delineation of the protective role of pDCs in AD, demonstrating that they restrain pathogenic Th2 inflammation and promote epidermal barrier repair, primarily through the IFN-α/IFNAR1 signaling axis.

In conditions such as systemic lupus erythematosus or alopecia areata (12, 13), pDCs can drive pathogenic inflammation, whereas in settings like skin wound healing and asthma, they exhibit anti-inflammatory and tissue-repair functions (10, 11). Our data align with the latter scenario, revealing a suppressive role for pDCs in AD. We observed a significant increase in pDCs in the peripheral blood of AD patients and dynamic recruitment of pDCs to lesional skin and draining lymph nodes in the MC903-induced AD mouse model, suggesting their active involvement in the disease process.

Functionally, expanding pDCs *in vivo* with FLT3L markedly alleviated AD-like inflammation, reducing clinical severity, Th2 cytokine levels, and serum IgE, while enhancing the expression of key epidermal barrier proteins. Conversely, specific depletion of pDCs with the 120G8 antibody exacerbated disease severity and amplified Th2 responses. These complementary gain- and loss-of-function experiments robustly establish pDCs as a negative regulatory component in AD pathogenesis.

Crucially, the protective effect of pDCs was abrogated in IFNAR1-deficient mice. FLT3L-mediated pDC expansion failed to mitigate inflammation or restore barrier protein expression in the absence of IFNAR1 signaling. This definitively shows that the immunosuppressive function of pDCs in AD is dependent on the IFN-α/IFNAR1 axis. Our findings are consistent with prior studies indicating that impaired pDC function or early-life deficiency is associated with exacerbated allergic inflammation (18). The sustained activation of cDC2 observed in our model, alongside pDC dynamics, supports a potential regulatory interplay where pDCs may act to restrain cDC2-driven Th2 polarization.

Beyond dampening immunity, pDC expansion also promoted the expression of structural proteins (FLG, IVL, KRT1), indicating a direct or indirect role in barrier repair (19). This effect may stem from IFN-α–mediated modulation of keratinocytes, suggesting a dual mechanism of action.

The recent success of JAK inhibitors (e.g., abrocitinib, upadacitinib) in treating moderate-to-severe AD highlights the central role of the JAK-STAT signaling pathway in AD pathogenesis. Our findings demonstrating the protective role of the IFN-α/IFNAR1 axis may initially seem counterintuitive, as JAK inhibitors broadly suppress cytokine signaling, including type I interferon signaling. However, we propose a nuanced interpretation: while acute pDC-derived IFN-α appears to limit Th2 inflammation in our model, chronic, dysregulated IFN signaling may contribute to epidermal barrier dysfunction and immune activation. We speculate that pDC-targeted therapies might synergize with JAK inhibitors by restoring immune homeostasis, or that JAK inhibitors may paradoxically enhance pDC function by reducing suppressive signals. Further studies are needed to explore these complex interactions.”

This study has limitations. The MC903 model primarily reflects acute, Th2-skewed inflammation, and further validation in chronic or humanized models is warranted. The functional state of human pDCs in AD also requires deeper characterization using single-cell technologies.

In conclusion, our data establish a previously underappreciated protective axis in AD: pDCs - IFN-α - IFNAR1 signaling - suppression of Th2 immunity and enhancement of barrier integrity. By moving beyond correlative human data to provide direct *in vivo* functional and mechanistic evidence, this work positions the pDC–type I interferon pathway as a potential foundation for novel therapeutic strategies in AD.

## Data Availability

The data presented in the study are deposited in the GSA repository, accession site https://ngdc.cncb.ac.cn/gsa-human/browse/HRA001797.
